# Are there differences in the quality of the diet of working and stay-at-home women?

**DOI:** 10.11606/S1518-8787.2018052000104

**Published:** 2018-04-20

**Authors:** Daniela de Assumpção, Caroline Senicato, Regina Mara Fisberg, Ana Maria Canesqui, Marilisa Berti de Azevedo Barros

**Affiliations:** I Universidade Estadual de Campinas. Faculdade de Ciências Médicas. Departamento de Saúde Coletiva. Campinas, SP, Brasil; II Universidade de São Paulo. Faculdade de Saúde Pública. Departamento de Nutrição. São Paulo, SP, Brasil

**Keywords:** Women, Women, Working, Healthy Diet, Socioeconomic Factors, Health Inequalities, Diet Surveys, Mulheres, Mulheres Trabalhadoras, Fatores Socioeconômicos, Dieta Saudável, Desigualdades em Saúde, Inquéritos sobre Dietas

## Abstract

**OBJECTIVE:**

To verify whether there is an association between the quality of the diet and the inclusion of women in the labor market and whether the education level would modify this association. We have analyzed the differences according to education level and evaluated whether the insertion or not in the market modifies the association between the quality of the diet and education level.

**METHODS:**

This is a cross-sectional population-based study that has used data from the Campinas Health Survey (2008 *ISACamp*). We have evaluated the diet of 464 women, aged 18 to 64 years, using the Brazilian Healthy Eating Index – Revised. We have estimated the means of the total score and index components using simple and multiple linear regression.

**RESULTS:**

We have observed no difference in the quality of diet of working and stay-at-home women. The analysis stratified by education level showed a lower intake of fruits among stay-at-home women in the segment of lower education level, in relation to working women. Among all women, a lower education level was associated with lower overall quality of the diet, higher intake of sodium, and lower intake of fruits, vegetables, whole grains, milk, and saturated fat. On the other hand, the inclusion in the labor market changed the effect of the education level on the quality of the diet. In the stay-at-home stratum, a low education level was associated with poorer quality of the diet and lower consumption of fruits, dark green and orange vegetables, and whole grains. Among the working women, a low education level was associated with higher intake of sodium and lower intake of vegetables, whole grains, and milk and dairy products.

**CONCLUSIONS:**

The results show inequities in the profile of food in relation to education level and inclusion in the labor market, which shows the relevance of public policies that increase the access to education and provide guidance on a healthy diet.

## RESUMO

**OBJETIVO:**

Verificar se existe associação entre qualidade da dieta e inserção das mulheres no mercado de trabalho e se o nível de escolaridade modificaria essa associação; analisar as diferenças segundo escolaridade; e avaliar se a inserção ou não no mercado modifica a associação entre qualidade da dieta e escolaridade.

**MÉTODOS:**

Trata-se de estudo transversal de base populacional, que utilizou dados do Inquérito de Saúde de Campinas (*ISACamp* 2008). A alimentação de 464 mulheres, de 18 a 64 anos, foi avaliada por meio do Índice de Qualidade da Dieta Revisado. Foram estimadas as médias do escore total e dos componentes do índice com o uso de regressão linear simples e múltipla.

**RESULTADOS:**

Nenhuma diferença foi observada entre qualidade da dieta de donas de casa e trabalhadoras remuneradas. A análise estratificada por escolaridade mostrou menor ingestão de frutas entre as donas de casa no segmento de pior escolaridade, em comparação às trabalhadoras remuneradas. Entre as mulheres, a menor escolaridade esteve associada à pior qualidade global da dieta e à maior ingestão de sódio e menor ingestão de frutas, vegetais, grãos integrais, leite e gordura saturada. Em contraste, a inserção no mercado de trabalho modificou o efeito da escolaridade sobre a qualidade da dieta. No estrato de donas de casa, a baixa escolaridade foi associada à pior qualidade da dieta e ao menor consumo de frutas, vegetais verde-escuros e alaranjados e grãos integrais. Entre as trabalhadoras remuneradas, a baixa escolaridade mostrou-se associada à ingestão maior de sódio e menor de vegetais, cereais integrais e leite e laticínios.

**CONCLUSÕES:**

Os resultados expõem iniquidades no perfil alimentar em relação à escolaridade e à inserção no mercado de trabalho, sinalizando a relevância de políticas públicas que ampliem o acesso à educação e à orientação sobre dieta saudável.

## INTRODUCTION

In the last decades of the twentieth century, economic, demographic, social, and political changes have increased the access of women to education and the labor market^1^. According to the 2010 Population Census, 55% of the economically active population in Brazil consisted of women, of whom 19.2% had complete higher education^2^.

On average, the main job demands approximately 36 hours per week for women and 42 hours for men; however, women spend 21 hours on household chores, which is more than twice the time spent by men^3^. Given the need to reconcile professional functions with those related to the care of the family and the home, women who work outside the house dedicate less time to prepare food^4^ ^–^ ^7^.

A cross-sectional study conducted in Minnesota, USA, with 3,709 parents or guardians of adolescents, has shown that women working full-time spent 8.8 hours per week cooking, a significantly shorter time in relation those who worked part-time (10.1 h) or who were unemployed (11.5 h)^6^.

The Brazilian traditional diet pattern has undergoing profound changes, in which fresh or minimally processed food, such as rice, beans, and cassava and the meals prepared from them, is being replaced by ready-to-consume food products^8^.

The finding on these inadequate food changes leads us to question whether the participation of women in the labor market would have contributed to these transformations. This discussion should consider a broad set of factors that contributed to changes in the food pattern. Among them, we can highlight the conformation of a food system based on monocultures that are transformed into food products by the industry and the action of transnational food companies in the national market, disseminating products of poor nutritional quality in all social strata and investing in marketing strategies that create “needs” in response to a daily life that requires time saving, practicality, satiety, and pleasure^9^ ^,^ ^10^. Another relevant issue is the lack of distribution of domestic activities related to food. Every family member should develop and share culinary skills so that this task is not attributed solely to females^8^.

The quality of the diet can be assessed and monitored using indexes, which bring together in a single measure a set of components based on food, nutrients, and culinary ingredients with proved health implications, such as whole grains, vegetables, saturated fat, and sodium^11^. The Brazilian Healthy Eating Index – Revised (BHEI-R) is an instrument adapted and validated for the Brazilian population, which allows the diagnosis of the level of quality of the diet of individuals or groups, based on nutritional recommendations^12^ ^,^ ^13^.

Considering the significant female participation in the workforce, the influence of women on family nutrition, the unfavorable changes in food culture, and the importance of adequate diet for the prevention of health problems, the objectives of this study were: to verify whether there is a distinction in the quality of the diet of working and stay-at-home women and whether the education level modifies these differences, to verify whether there are differences according to education level, and to evaluate whether or not being included in the labor market modifies the association between quality of the diet and education level.

## METHODS

This is a cross-sectional population-based study that used data from the Campinas Health Survey (2008 *ISACamp*), carried out from February 2008 to March 2009.

The study aimed to analyze the life and health conditions of three age groups: adolescents aged 10 to 19 years, adults aged 20 to 59 years, and older adults aged 60 years and over. The sample size was defined considering the estimation of a ratio of 0.50 (corresponding to maximum variability), sampling error between four and five percentage points, 95% confidence interval, and a design effect of two, resulting in 1,000 individuals for each age group. Predicting an 80% response rate, we corrected the sample size to 1,250 individuals.

The 2008 *ISACamp* sample was obtained by probabilistic sampling, by clusters, and in two stages: census tract and household. In the first stage, after ordering the census tracts by the percentage of heads of families with university education, 50 tracts were systematically draw for the urban area of the city of Campinas, state of São Paulo, Brazil, with probability proportional to the number of households. In the second stage, a systematic sampling of households was carried out.

Independent household samples were selected for each age group, corresponding to 2,150 interviews for adolescents, 700 for adults, and 3,900 for older adults. All the residents of the household that belonged to the domain drawn were interviewed. Further information on the sampling plan is available in Alves^14^.

In this study, we analyzed only data from female adults in the age group of 18 to 64 years. This group is used by international and national studies to classify women of productive age^15^ ^,^ ^16^.

Data on the demographic, socioeconomic, and health conditions, health-related behaviors, and food habits were obtained from a structured previously tested questionnaire, which was applied by trained and supervised interviewers. Food consumption was estimated by a 24-hour recall (24HR), a method that consists of the collection and quantification of all types of food and beverages consumed the day before the interview^17^.

The variables analyzed here were:

- Brazilian Healthy Eating Index – Revised (BHEI-R)^12^. The instrument has twelve components classified into adequacy (total fruits, whole fruits, total vegetables and legumes, dark green and orange vegetables and legumes, total grains, whole grains, milk and dairy products, meats, eggs and legumes, oils) and moderation (sodium, saturated fat, SoFAAS – which evaluates the energy percentage from solid fats, alcohol, and added sugar) (Box).

**Box:** Criteria of the score of the components of the BHEI-R.

Components of the BHEI-R	Score range (minimum to maximum)	Criteria of the minimum score	Criteria of the maximum score
Adequacy (higher score means higher consumption)			
1. Total fruits^a^	0 to 5	No consumption	1.0 portion/1,000 kcal
2. Whole fruits	0 to 5	No consumption	0.5 portion/1,000 kcal
3. Total vegetables	0 to 5	No consumption	1.0 portion/1,000 kcal
4. Dark green and orange vegetables	0 to 5	No consumption	0.5 portion/1,000 kcal
5. Total grains	0 to 5	No consumption	2.0 portions/1,000 kcal
6. Whole grains	0 to 5	No consumption	1.0 portion/1,000 kcal
7. Milk and dairy products	0 to 10	No consumption	1.5 portion/1,000 kcal
8. Meat and eggs^b^	0 to 10	No consumption	1.0 portion/1,000 kcal
9. Oils^c^	0 to 10	No consumption	0.5 portion/1,000 kcal
Moderation (higher score means lower consumption)			
10. Saturated fat	0 to 10	≥ 15% of TEV	≤ 7% of TEV
11. Sodium	0 to 10	≥ 2.0 g/1,000 kcal	≤ 0.75 g/1,000 kcal
12. SoFAAS	0 to 20	≥ 35% of TEV	≤ 10% of the TEV
Total BHEI-R	0 to 100		

Source: Previdelli et al.^12^ (2011)

BHEI-R: Brazilian Healthy Eating Index – Revised; TEV: total energy value; SoFAAS: calories from solid fat, alcohol, and added sugar

a Represents the consumption of fruit in the form of natural juice.

b Legumes were excluded from the component of meats and eggs.

c Includes oil and fish fat.

Each component receives a specific score, ranging from zero to five, or from zero to ten, or from zero to twenty. The minimum score is attributed to the non-consumption of the adequacy components (1 to 9) or consumption above the recommended limit of the moderation components (10 to 12). The maximum score is given by reaching or exceeding the recommended value of intake for each component. Intermediate values of intake are given proportional scores. The sum of the scores of the components results in the total BHEI-R, which can reach up to 100 points (Box).

Data from a 24-hour recall were used to calculate the BHEI-R. After confirming the quality of the filling, the recalls were quantified by a nutritionist to convert the information from home measures into grams or milliliters. The quantification was done using tables of home measurements^18^ ^,^ ^19^, food labels, and consumer services.

The software Nutrition Data System for Research, 2007 version (University of Minnesota), was used to calculate the nutrients of the food ingested. The recalls with an energy value of less than 800 kcal or more than 3,500 kcal went through a consistency analysis, with a review of all content.

According to the method proposed by the 2005 Healthy Eating Index, the BHEI-R transfers the energy from the legumes to the component of *meat and eggs*, if the consumption of these types of food falls below the recommended value. If the component of *meat and eggs* reaches the maximum score and there is energy left for legumes, this surplus goes to two other components: *total vegetables* and *dark green and orange vegetables*, respectively. In this study, we decided to exclude the legumes from the BHEI-R method in order not to overestimate the scores of the vegetables, as the Brazilian population consumes more legumes than the North American population. Another reason for the exclusion refers to the incompatibility of beans and meats in terms of nutritional profile, quality, and utilization of protein.

- Inclusion in the labor market: categorized into working and stay-at-home women. In the group of working women, we included women who performed some paid activity at the time of the research, those who were temporarily away from work because of illness, and those who were retired and continued to have a paid work. We considered as stay-at-home women those who had no paid work and who reported to be stay-at-home women. In this study, we excluded women who were unemployed (n = 38), retired (n = 101), and students (n = 34) because they were not included in the labor market or because they received retirement or unemployment benefits.
- Education level: evaluated in years of study and categorized into zero to eight years and nine years or more.
- Demographic and socioeconomic variables: age group (years), education level, monthly family income *per capita* (minimum wage), self-reported race, marital status, number of children, number of persons in the household, and presence of housekeeper.

In this study, the dependent variables were the scores of the total DQI-R and of each component, and the independent variables were the inclusion in the labor market and the education level of the women. We used the demographic and socioeconomic variables to describe the social characteristics of the working and stay-at-home women.

The associations between the demographic and socioeconomic variables and occupation were determined by the Rao-Scott chi-square test, with a 5% significance level. The means of total BHEI-R and of each component were estimated by simple and multiple linear regression, considering a 5% significance level. The multiple model was adjusted for age, income, marital status, and number of children. The energy (kcal) did not enter the model as an adjustment variable because the BHEI-R was produced at a density of 1,000 kcal.

We analyzed the data in the *svy* module of the Stata 11.0 program, considering the study sampling design. This research was approved by the Research Ethics Committee of the Universidade Estadual de Campinas and the National Commission for Research Ethics (CEP/CONEP system), under CAAE 37303414.4.0000.5404.

## RESULTS

We excluded 235 women from the analysis: 160 hypertensive, 52 diabetic, and 23 pregnant women. Thus, the study population consisted of 464 women aged 18 to 64 years. Mean age was 36.3 years (95%CI 35.1–37.5) for all women evaluated, 35.3 years (95%CI 33.9–36.6) for working women, and 40.3 years (95%CI 37.7–42.8) for stay-at-home women.

Table 1 shows that most full-time stay-at-home women are older, married, with lower education level and family income *per capita*, and with three or more children.

**Table 1:** Demographic and socioeconomic characteristics according to inclusion in the labor market of women aged 18 to 64 years. Campinas Health Survey (2008 *ISACamp*).

Variable		Total		Working women		Stay-at-home women		p^a^
		n	%	n	%	n	%	
	Total	464		316		148		
Age group (years)								0.0001
	18–29	151	32.3	116	34.0	35	27.9	
	30–39	102	28.6	82	32.6	20	18.9	
	40–49	85	23.2	58	22.6	27	24.9	
	50–64	126	15.9	60	10.8	66	28.3	
Education level (years of study)								< 0.0001
	0–8	198	39.2	102	30.3	96	60.9	
	9 or more	266	60.8	214	69.7	52	39.1	
Family income *per capita* (MW^b^)								0.0001
	< 1	180	38.4	106	31.5	74	55.3	
	≥ 1	284	61.6	210	68.5	74	44.7	
Race (self-reported)^c^								0.4057
	White	344	76.1	232	74.7	112	79.6	
	Black	28	6.6	24	7.9	4	3.6	
	Brown	88	17.3	57	17.4	31	16.8	
Marital status								< 0.0001
	Married	220	47.8	123	41.0	97	64.1	
	Living together/Cohabitating	68	16.7	45	16.0	23	18.5	
	Divorced/Separated/Widowed	53	10.2	36	10.2	17	10.1	
	Single	123	25.3	112	32.8	11	7.3	
Number of children								< 0.0001
	Zero	132	28.6	125	38.4	7	4.9	
	1	98	23.2	68	23.7	30	22.0	
	2	118	25.4	64	20.9	54	36.1	
	3 or more	116	22.8	59	17.0	57	37.0	
Number of persons in the household								0.0584
	1 to 2	122	22.2	88	25.4	34	14.6	
	3 to 4	213	49.6	146	49.1	67	50.6	
	5 or more	129	28.2	82	25.5	47	34.8	
Housekeeper								0.3707
	No	417	90.3	282	89.4	135	92.5	
	Yes	47	9.7	34	10.6	13	7.5	

a Rao-Scott chi-square test.

b Minimum wage (MW) valid at the time of the research: January to April/2008: R$415.00; May/2008 to April/2009: R$450.00.

c Four women who self-reported as yellow were excluded.

We found no differences in the scores of the components and overall quality of the diet among working and stay-at-home women (Table 2).

**Table 2:** Mean scores and beta coefficients of the components of the BHEI-R of working and stay-at-home women aged 18 to 64 years. Campinas Health Survey (2008 *ISACamp*).

Components of the BHEI-R	Means (95%CI)		Beta coefficients (p)	
	Working women^a^	Stay-at-home women	Crude	Adjusted^b^
Total fruits	2.11 (1.77–2.46)	1.77 (1.34–2.19)	-0.35 (0.135)	-0.35 (0.110)
Whole fruits	2.12 (1.72–2.52)	2.09 (1.59–2.59)	-0.03 (0.901)	0.03 (0.891)
Total vegetables	3.20 (2.99–3.42)	2.82 (2.38–3.26)	-0.38 (0.050)	0.0004 (0.998)
Dark green and orange vegetables	1.59 (1.35–1.83)	1.16 (0.80–1.53)	-0.43 (0.028)	-0.25 (0.277)
Total grains	4.63 (4.52–4.74)	4.65 (4.47–4.83)	0.02 (0.832)	0.04 (0.658)
Whole grains	0.26 (0.12–0.40)	0.27 (0.07–0.46)	0.01 (0.928)	0.003 (0.981)
Milk and dairy products	4.72 (4.18–5.27)	4.35 (3.69–5.00)	-0.38 (0.361)	0.23 (0.529)
Meat and eggs	8.27 (7.97–8.57)	8.21 (7.71–8.70)	-0.06 (0.810)	-0.11 (0.705)
Sodium	2.42 (2.07–2.76)	2.11 (1.67–2.54)	-0.31 (0.218)	-0.22 (0.448)
Oils	8.48 (8.04–8.92)	9.05 (8.51–9.59)	0.57 (0.098)	0.38 (0.334)
Saturated fat	5.67 (5.19–6.16)	6.44 (5.71–7.17)	0.77 (0.074)	0.58 (0.201)
SoFAAS	9.31 (8.57–10.05)	10.51 (9.31–11.70)	1.20 (0.077)	0.68 (0.322)
Total BHEI-R	52.79 (51.24–54.34)	53.42 (50.97–55.86)	0.63 (0.590)	1.03 (0.436)

BHEI-R: Brazilian Healthy Esting Index – Revised; SoFAAS: calories from solid fat, alcohol, and added sugar

a Reference category used for comparison.

b Adjusted for age, education level, income, marital status, and number of children.

When analyzing the inclusion in the labor market stratified by education level, the only difference we found was that the scores for total fruits and whole fruits were lower in stay-at-home women among the women with up to eight years of education, which meant lower consumption of fruits and natural juices (Table 3).

**Table 3:** Mean scores and beta coefficients of the components of the BHEI-R, according to the inclusion in the labor market and the education level of women aged 18 to 64 years. Campinas Health Survey (2008 *ISACamp*).

Components of the BHEI-R	Adjusted analyses^a^					
	Up to 8 years			9 years or more		
	Working women^b^	Stay-at-home women	β (p)	Working women^b^	Stay-at-home women	β (p)
Total fruits	1.99	1.42	-0.81 (0.004)	2.17	2.31	0.25 (0.567)
Whole fruits	1.90	1.52	-0.71 (0.015)	2.21	2.97	0.82 (0.081)
Total vegetables	2.49	2.55	-0.01 (0.974)	3.52	3.24	-0.01 (0.977)
Dark green and orange vegetables	1.31	0.86	-0.52 (0.053)	1.71	1.64	0.12 (0.786)
Total grains	4.56	4.67	0.10 (0.442)	4.66	4.62	-0.10 (0.452)
Whole grains	0.16	0.10	-0.14 (0.186)	0.30	0.54	0.20 (0.430)
Milk and dairy products	3.48	3.82	0.27 (0.636)	5.27	5.18	0.03 (0.957)
Meat and eggs	8.53	8.09	-0.57 (0.154)	8.16	8.38	0.28 (0.475)
Sodium	2.12	1.88	-0.09 (0.807)	2.54	2.46	-0.38 (0.364)
Oils	8.64	9.08	0.54 (0.241)	8.40	9.00	0.36 (0.429)
Saturated fat	6.34	6.74	0.17 (0.750)	5.39	5.98	1.12 (0.123)
SoFAAS	9.90	11.40	0.86 (0.249)	9.05	9.11	0.24 (0.807)
Total BHEI-R	51.44	52.13	-0.91 (0.593)	53.37	55.42	2.93 (0.226)

BHEI-R: Brazilian Healthy Eating Index – Revised; SoFAAS: calories from solid fat, alcohol, and added sugar

a Adjusted for age, income, marital status, and number of children.

b Reference category used for comparison.

Table 4 shows the association between education level and the quality of the diet. Women with lower education level had worse overall quality of the diet, higher intake of sodium, and lower intake of fruits, total vegetables, whole grains, milk and dairy products, and saturated fat.

**Table 4:** Mean scores and beta coefficients of the components of the BHEI-R, according to the education level of women aged 18 to 64 years. Campinas Health Survey (2008 *ISACamp*).

Components of the BHEI-R	Means (95%CI)		Beta coefficients (p)	
	9 years or more^a^	Up to 8 years	Crude	Adjusted^b^
Total fruits	2.21 (1.83–2.60)	1.76 (1.38–2.14)	-0.45 (0.078)	-0.57 (0.035)
Whole fruits	2.41 (1.98–2.83)	1.75 (1.31–2.19)	-0.65 (0.017)	-0.83 (0.002)
Total vegetables	3.41 (3.17–3.65)	2.63 (2.25–3.01)	-0.78 (0.001)	-0.73 (0.005)
Dark green and orange vegetables	1.66 (1.36–1.97)	1.27 (0.93–1.60)	-0.39 (0.084)	-0.28 (0.250)
Total grains	4.63 (4.51–4.76)	4.61 (4.46–4.76)	-0.02 (0.825)	-0.02 (0.873)
Whole grains	0.38 (0.22–0.54)	0.12 (0.03–0.21)	-0.26 (0.004)	-0.36 (0.002)
Milk and dairy products	5.29 (4.77–5.80)	3.66 (3.11–4.22)	-1.62 (0.000)	-1.66 (0.000)
Meat and eggs	8.04 (7.67–8.41)	8.32 (7.94–8.69)	0.27 (0.283)	0.19 (0.554)
Sodium	2.63 (2.28–2.99)	2.00 (1.66–2.34)	-0.64 (0.007)	-0.72 (0.005)
Oils	8.41 (8.00–8.82)	8.92 (8.41–9.43)	0.51 (0.111)	-0.04 (0.911)
Saturated fat	5.61 (5.10–6.12)	6.59 (6.00–7.18)	0.98 (0.012)	0.98 (0.030)
SoFAAS	9.03 (8.31–9.76)	10.88 (9.87–11.88)	1.84 (0.003)	0.86 (0.205)
Total BHEI-R	53.73 (52.10–55.36)	52.52 (50.53–54.50)	-1.21 (0.300)	-3.17 (0.013)

BHEI-R: Brazilian Healthy Eating Index – Revised; SoFAAS: calories from solid fat, alcohol, and added sugar

a Reference category used for comparison.

b Adjusted for age, income, marital status, and number of children.

Table 5 shows that stay-at-home women with low education level presented lower overall quality of the diet and lower intake of whole fruits, dark green and orange vegetables, and whole grains. Among working women, those who had up to eight years of education had higher intake of sodium and lower intake of total vegetables, whole grains, milk and dairy products, than the more educated women.

**Table 5:** Mean scores and beta coefficients of the components of the BHEI-R, according to education level of working and stay-at-home women aged 18 to 64 years. Campinas Health Survey (2008 *ISACamp*).

Components of the BHEI-R	Adjusted analyses^a^					
	Stay-at-home women			Working women		
	Up to 8 years	9 years or more^b^	β (p)	Up to 8 years	9 years or more^b^	β (p)
Total fruits	1.42	2.31	-0.88 (0.066)	1.99	2.17	-0.42 (0.235)
Whole fruits	1.52	2.97	-1.60 (0.001)	1.90	2.21	-0.56 (0.153)
Total vegetables	2.55	3.24	-0.79 (0.118)	2.49	3.52	-0.94 (0.002)
Dark green and orange vegetables	0.86	1.64	-1.04 (0.014)	1.31	1.71	-0.07 (0.827)
Total grains	4.67	4.62	0.12 (0.505)	4.56	4.66	-0.14 (0.314)
Whole grains	0.10	0.54	-0.67 (0.040)	0.16	0.30	-0.30 (0.009)
Milk and dairy products	3.81	5.18	-1.29 (0.115)	3.48	5.27	-1.84 (0.001)
Meat and eggs	8.09	8.38	-0.82 (0.089)	8.53	8.16	0.66 (0.084)
Sodium	1.88	2.46	-0.09 (0.799)	2.12	2.54	-0.75 (0.028)
Oils	9.01	8.99	-0.03 (0.947)	8.64	8.40	-0.18 (0.689)
Saturated fat	6.74	5.98	0.83 (0.319)	6.34	5.39	0.86 (0.164)
SoFAAS	11.40	9.11	0.43 (0.694)	9.90	9.04	0.12 (0.889)
Total BHEI-R	52.13	55.42	-5.83 (0.026)	51.44	53.37	-3.54 (0.075)

BHEI-R: Brazilian Healthy Eating Index – Revised; SoFAAS: calories from solid fat, alcohol, and added sugar

a Adjusted for age, income, marital status, and number of children.

b Reference category used for comparison.

## DISCUSSION

The results of this study expose the presence of iniquities in the living conditions between working and stay-at-home women. Stay-at-home women are older, married, poorer, with more children, and with lower education level. Research studies indicate that stay-at-home women with lower education levels and family income had worse quality of life related to health^20^ and higher prevalence of chronic non-communicable diseases, mental disorders, and depression^15^ ^,^ ^21^ ^,^ ^22^. However, the benefits of employment depend on the position held at work and the socioeconomic level of the women^16^ ^,^ ^23^ ^,^ ^24^.

Regarding the quality of the diet according to the female inclusion in the labor market, we found no difference in the initial analysis. The lack of disparities in the diet was confirmed when we compared working and stay-at-home women with the same education level, and we could only note that the intake of fruits was lower among stay-at-home women than among working women in the stratum of lower education level. Research studies that have analyzed data from 187 countries indicate that the consumption of healthy food, including fruits and fruit juices, is directly related to the income level of the countries^25^ ^,^ ^26^. In Brazil, the Household Budget Survey (2008–2009 POF) shows that the consumption of fruits and natural juices increases with increasing family income^27^. This result is consistent with that obtained by a study that has evaluated the quality of the diet of the population of Campinas, state of São Paulo, Brazil, which has verified increasing score for fruits with increasing education level of the head of the family^28^. Some hypotheses could partially explain the higher intake of fruits among working women only in the lower education stratum. It is known that more than 19.5 million Brazilian workers are served by the Worker's Food Program (PAT), whose menus must contain at least a portion of fruit in each meal provided^29^ ^,^ ^30^. Buffet restaurants, which is a model spread throughout the country, offer a wide variety of affordable food, including fruits and natural juices, enabling persons to make healthy food choices^8^ ^,^ ^31^.

Studies that analyze the profile of food of women according to their participation in the labor market are scarce and present controversial results. A qualitative study carried out in Viçosa, state of Minas Gerais, Brazil, has found that factors such as age, income, family support, occupation, and health concern modified the diet more than being included in the labor market^7^. Analyzing 1,555 Australian women aged 18 to 65, Thornton et al.^32^ have found that the work outside the house did not modify the consumption of fruits, vegetables, and fast foods; however, the supply of healthy food near the workplace was associated with a higher intake of fruits and vegetables. On the other hand, in the study by Bauer et al.^6^, women who worked full-time ate less fruits and vegetables, had fewer family meals, and gave their children less information about a healthy diet compared to the women who did not work or who worked part-time.

In this research, education level clearly differentiated the quality of the dietary pattern of the women. Lower education level was associated with poorer quality of the diet, as well as higher intake of sodium and lower intake of fruits, vegetables, whole grains, and saturated fat. Other researchers have detected an association between the increase in education level and the improvement of the overall quality of the diet, from the greater consumption of healthy food^28^ ^,^ ^33^. The results of the 2008–2009 POF show that the low-income family segments have a lower consumption of food high in saturated fats, such as meats, sausages, milk, cheese, and butter^27^. Higher intake of sodium in the low education segment is consistent with the results of a study that has observed higher household availability of sodium and food high in sodium among economically disadvantaged persons^34^.

Although the education level can positively affect the diet, a study carried out in Campinas, state of São Paulo, Brazil, has found that even in the stratum of 12 years or more of education, the score of the total BHEI-R was very low (54.4 points on a scale from zero to 100), which shows the importance of promoting healthy eating habits in the entire population^28^. Education encourages assertiveness, autonomy, and social participation of the women, which is essential to promote physical and mental health, in addition to more appropriate food choices^35^. In a population of low-income women, Lins et al.^36^ have found that the consumption of fruits and vegetables and the report of having a healthy diet were more prevalent among those with higher income and education. A qualitative study has observed that higher education level and income differentiated the food repertoire of the meals of the families studied, providing the inclusion of fruits, vegetables, cheese, yogurt, whole-wheat bread, and different types of meat^37^.

The inclusion in the labor market had a modifying effect on the association between the quality of the diet and education level. Among the working and stay-at-home women, higher education level appears to have increased the knowledge and opportunity for women to adhere to a healthier diet. We found no study developed under this perspective in the literature, which indicates the need to investigate this subject.

The Food Guide for the Brazilian Population (2014) warns that the loss of the custom of transmitting culinary skills between generations has contributed to the lack of autonomy and experience in food preparation in younger persons, who become increasingly dependent on ready-made or almost ready-made food^8^. Faced with the change in the transmission of culinary knowledge, Diez-Garcia and Castro^37^ indicate the importance of interventions aimed at the development of the aptitude and autonomy in the preparation of food and the realization of meals at home as strategies to improve the quality of the diet of families. The loss of the value in cooking is reinforced by food advertising campaigns that spread the idea that modern women do not have time to lose to prepare food, disregarding the social and symbolic meaning of eating and cooking^8^ ^,^ ^10^.

Canesqui^38^ shows in an ethnographic study with low-income, urban working families the changes in the transmission and use of culinary knowledge between two generations of women, noting that the generation of older women, who had a rural past, early socialized their daughters as future stay-at-home women, including the transmission of this knowledge, incorporating them effectively in the household chores of cooking food. The second generation of women, with the accumulation of experience of living in the city, on the contrary, socialized late their daughters in these tasks, did not delegate the cooking activity to them, generally giving them the work of cleaning the food and washing the household appliances and kitchen floors. This latter generation spared their adolescent daughters from household chores, ideologically devaluing the unpaid domestic work among the activities of the women, aspiring for them the reward for their investments in their education, preparation, and qualification for paid work.

The evaluation of the results of this study needs to consider some limitations. One of them refers to the use of a single 24-hour recall, hindering the analysis of the usual intake of an individual given the great variability in food consumption^17^. However, we can estimate a mean intake for the population when the recall is applied on a population basis and it considers the different days of the week and months of the year, as in the 2008 *ISACamp* survey^39^. The cross-sectional study hinders the interpretation of the associations found as resulting from cause and effect relationships. The *ISACamp* is not a specific nutrition research, therefore it does not present detailed information related to food consumption. The knowledge about the frequency and types of food consumed at home and away from home and who buys and prepares the food could broaden the discussion of the results found in this study.

We can conclude that the quality of the diet of the women is modified by the inclusion in the labor market and the education level. Therefore, social policies are needed to increase the access of women to the educational system, as well as actions that promote healthy eating habits in the entire population.
